# Aquaporin 2 Mutations in *Trypanosoma brucei gambiense* Field Isolates Correlate with Decreased Susceptibility to Pentamidine and Melarsoprol

**DOI:** 10.1371/journal.pntd.0002475

**Published:** 2013-10-10

**Authors:** Fabrice E. Graf, Philipp Ludin, Tanja Wenzler, Marcel Kaiser, Reto Brun, Patient Pati Pyana, Philippe Büscher, Harry P. de Koning, David Horn, Pascal Mäser

**Affiliations:** 1 Swiss Tropical and Public Health Institute, Basel, Switzerland; 2 University of Basel, Basel, Switzerland; 3 Institut National de Recherche Biomédicale, Kinshasa-Gombe, Democratic Republic of the Congo; 4 Department of Biomedical Sciences, Institute of Tropical Medicine, Antwerp, Belgium; 5 Institute of Infection, Immunity and Inflammation, College of Medical, Veterinary and Life Sciences, University of Glasgow, Glasgow, United Kingdom; 6 Biological Chemistry and Drug Discovery, College of Life Sciences, University of Dundee, Dundee, United Kingdom; Makerere University, Uganda

## Abstract

The predominant mechanism of drug resistance in African trypanosomes is decreased drug uptake due to loss-of-function mutations in the genes for the transporters that mediate drug import. The role of transporters as determinants of drug susceptibility is well documented from laboratory-selected *Trypanosoma brucei* mutants. But clinical isolates, especially of *T. b. gambiense*, are less amenable to experimental investigation since they do not readily grow in culture without prior adaptation. Here we analyze a selected panel of 16 *T. brucei* ssp. field isolates that (i) have been adapted to axenic *in vitro* cultivation and (ii) mostly stem from treatment-refractory cases. For each isolate, we quantify the sensitivity to melarsoprol, pentamidine, and diminazene, and sequence the genomic loci of the transporter genes *TbAT1* and *TbAQP2*. The former encodes the well-characterized aminopurine permease P2 which transports several trypanocides including melarsoprol, pentamidine, and diminazene. We find that diminazene-resistant field isolates of *T. b. brucei* and *T. b. rhodesiense* carry the same set of point mutations in *TbAT1* that was previously described from lab mutants. Aquaglyceroporin 2 has only recently been identified as a second transporter involved in melarsoprol/pentamidine cross-resistance. Here we describe two different kinds of *TbAQP2* mutations found in *T. b. gambiense* field isolates: simple loss of *TbAQP2*, or loss of wild-type *TbAQP2* allele combined with the formation of a novel type of *TbAQP2/3* chimera. The identified mutant *T. b. gambiense* are 40- to 50-fold less sensitive to pentamidine and 3- to 5-times less sensitive to melarsoprol than the reference isolates. We thus demonstrate for the first time that rearrangements of the *TbAQP2/TbAQP3* locus accompanied by *TbAQP2* gene loss also occur in the field, and that the *T. b. gambiense* carrying such mutations correlate with a significantly reduced susceptibility to pentamidine and melarsoprol.

## Author Summary

Human African Trypanosomiasis, or sleeping sickness, is a fatal disease restricted to sub-Saharan Africa, caused by *Trypanosoma brucei gambiense* and *T. b. rhodesiense*. The treatment relies on chemotherapy exclusively. Drug resistance in *T. brucei* was investigated mainly in laboratory-selected lines and found to be linked to mutations in transporters. The adenosine transporter TbAT1 and the aquaglyceroporin TbAQP2 have been implicated in sensitivity to melarsoprol and pentamidine. Mutations in these transporters rendered trypanosomes less susceptible to either drug. Here we analyze *T. brucei* isolates from the field, focusing on isolates from patients where melarsoprol treatment has failed. We genotype those isolates to test for mutations in *TbAQP2* or *TbAT1*, and phenotype for sensitivity to pentamidine and melarsoprol. Six *T. b. gambiense* isolates were found to carry mutations in *TbAQP2*. These isolates stemmed from relapse patients and exhibited significantly reduced sensitivity to pentamidine and melarsoprol as determined in cell culture. These findings indicate that mutations in *TbAQP2* are present in the field, correlate with loss of sensitivity to pentamidine and melarsoprol, and might be responsible for melarsoprol treatment failures.

## Introduction

The chemotherapy of human African trypanosomiasis (HAT, also known as sleeping sickness) currently relies on suramin or pentamidine for the first, haemolymphatic stage and on melarsoprol or eflornithine/nifurtimox combination therapy (NECT) for the second stage, when the trypanosomes have invaded the central nervous system (CNS) [1]. All five drugs have unfavorable pharmacokinetics and adverse effects. Melarsoprol is particularly toxic, causing severe encephalopathies in over 5% of the treated patients [2]. And yet, melarsoprol is the only treatment for late-stage *T. b. rhodesiense* infections. New and safer drugs are at various stages of (pre)clinical development, thanks largely to the Drugs for Neglected Diseases initiative (www.dndi.org). Two molecules that have successfully passed clinical Phase I trials are now being tested in patients: the nitroimidazole fexinidazole [3], [4] and the benzoxaborole SCYX-7158 [5], [6]. Both are orally available and cure 2^nd^ stage *T. b. brucei* infections in a mouse model [7]. However, until new drugs for HAT are on the market, the current ones – problematic as they are – need to be used in a sustainable way. This requires an understanding of the mechanisms of drug resistance.

The mechanisms of drug resistance in African trypanosomes have been studied in the lab for over 100 years [8]. Two observations were made recurrently, namely (i) reduced drug uptake by drug resistant trypanosomes [9]–[14] and (ii) cross-resistance between melarsoprol and pentamidine [15], [16]. Both phenomena were attributed to the fact that melarsoprol and pentamidine are taken up by trypanosomes via the same transporters, which appeared to be lacking in drug-resistant mutants. The first transporter identified was called P2 since it was one of two purine nucleoside transporters identified [17], [18]. It is encoded by the gene *TbAT1* for adenine/adenosine transporter 1 [19]. Homozygous genetic deletion of *TbAT1* in bloodstream-form *T. b. brucei* resulted in pentamidine and melarsoprol cross-resistance, albeit only by a factor of about 2.5 [20]. This weak phenotype, together with the fact that the *TbAT1^−/−^* mutants still exhibited saturable drug import [21], indicated that further transporters are involved in melarsoprol-pentamidine cross-resistance [16], [21], [22]. One such transporter was recently identified, the aquaglyceroporin TbAQP2 [23], [24]. Aquaporins and aquaglyceroporins belong to the major intrinsic protein (MIP) family and form channels that facilitate transmembrane transport of water and small non-ionic solutes such as glycerol and urea [25]. The three aquaporins of *T. brucei* (TbAQP1-3) are thought to physiologically function as osmoregulators and are involved in glycerol transport [26]. Aquaporins were described to mediate uptake of arsenite in mammalian cells [27] and in *Leishmania*, and loss of aquaporin function was implicated in heavy metal resistance [28]. Homozygous genetic deletion of *TbAQP2* in bloodstream-form *T. b. brucei* increased the IC_50_ towards melarsoprol and pentamidine by about 2- and 15- fold, respectively [24]. Moreover, a *T. b. brucei* lab mutant selected for high-level pentamidine resistance [21] carried a chimeric *TbAQP2* gene, where 272 nucleotides had been replaced by the corresponding sequence from a neighboring, very similar gene *TbAQP3* [24]. Differences in the *TbAQP2/TbAQP3* tandem locus on chromosome 10 were also observed between the reference genome sequences of *T. b. gambiense* DAL972 [29] and *T. b. brucei* TREU927 [23], [30]. They possess identical versions of *TbAQP2* but differ in *TbAQP3* [31]. More recent field isolates of *T. brucei* ssp. have so far not been genotyped regarding their *TbAQP2/TbAQP3* locus.

The genotypic status of *TbAT1*, located proximal to a telomere on chromosome 5 [32], has been more intensely investigated. Point mutations in *TbAT1* were described, both in selected lab strains and in clinical *T. brucei* ssp. isolates, which rendered the gene non-functional when expressed in yeast [19]. The occurrence of these mutations correlated to a certain degree with melarsoprol treatment failure in 2^nd^ stage *T. b. gambiense* HAT patients [33]–[36]. However, the relationship between polymorphisms in *TbAT1*, drug susceptibility, and treatment failure in patients is not fully resolved as the *TbAT1* mutant *T. b. gambiense* were not analyzed phenotypically. Such investigations are notoriously difficult since clinical *T. b. gambiense* isolates are hard to obtain (given the inaccessibility of HAT foci and the poor success rate of isolation and adaptation in rodents) and cannot readily be propagated in axenic culture. Here we concentrate on clinical *T. brucei* ssp. isolates from drug refractory cases that have been adapted to axenic *in vitro* cultivation, aiming to investigate whether mutations at the known melarsoprol and pentamidine transporter loci also occur in the field – and if so, whether such mutations are accompanied by loss of drug susceptibility.

## Materials and Methods

### *Trypanosoma brucei* ssp. isolates

The 16 analyzed isolates are described in Table 1 (origin) and Table 2 (clinical outcome). For more details on the recent isolates from the DRC please refer to Table S4 of Pyana et al (2011) [37]. All have previously been adapted to axenic cultivation. *T. b. brucei* and *T. b. rhodesiense* isolates were cultured in minimum essential medium (MEM) with Earle's salts with the addition of 0.2 mM 2-mercaptoethanol, 1 mM Na-pyruvate, 0.5 mM hypoxanthine, and 15% heat-inactivated horse serum as described by Baltz et al (1985) [38]. *T. b. gambiense* strains were cultured in IMDM medium supplemented according to Hirumi and Hirumi (1989) [39], plus 0.2 mM 2-mercaptoethanol, 15% heat-inactivated fetal calf serum and 5% human serum. The cultures were maintained under a humidified 5% CO_2_ atmosphere at 37°C and were subpassaged 3 times a week to ensure growth in the exponential (log) phase.

**Table 1:** Origin of the analyzed *T. brucei* isolates.

Isolate	Species	Origin	Reference
STIB 930	*Tbg*	Republic of Côte d'Ivoire, 1978	[49]
ITMAP 141267	*Tbg*	Democratic Republic of the Congo, 1960	[50]
STIB 756	*Tbg*	Liberia, 1981	[51]
STIB 891	*Tbg*	Uganda, 1995	[33]
DAL 870R	*Tbg*	Republic of Côte d'Ivoire, 1985	[52]
DAL 898R	*Tbg*	Republic of Côte d'Ivoire, 1985	[52]
K03048	*Tbg*	South Sudan, 2003	[53]
45 BT (MHOM/CD/INRB/2006/1)	*Tbg*	Democratic Republic of the Congo, 2006	[37]
130 BT (MHOM/CD/STI/2006/02)	*Tbg*	Democratic Republic of the Congo, 2006	[37]
349 BT (MHOM/CD/INRB/2006/16)	*Tbg*	Democratic Republic of the Congo, 2006	[37]
349 AT (MHOM/CD/INRB/2006/19)	*Tbg*	Democratic Republic of the Congo, 2006	[37]
40 AT (MHOM/CD/INRB/2006/07)	*Tbg*	Democratic Republic of the Congo, 2006	[37]
STIB 900	*Tbr*	Tanzania, 1982	[52]
STIB 871	*Tbr*	Uganda, 1994	[54]
STIB 940	*Tbb*	Somalia, 1985	[42], [55]
STIB 950	*Tbb*	Somalia, 1985	[41]

**Table 2:** Drug sensitivity (IC_50_ ± SD in nM), genotypic status of *TbAT1* and *TbAQP2*, and clinical outcome of melarsoprol treatment of the patients.

Isolate	MelB	Pentamidine	Diminazene	*TbAT1*	*TbAQP2*	Clinics
STIB 930	9.6±4.5	1.9±0.7	21.0±8.5	Ref	Ref	Cure
ITMAP 141267	15.0±8.1	8.3±3.4	9.9±4.4	WT	WT	Cure
STIB 756	6.2±1.1	1.3±0.7	24.7±7.9	WT	WT	Unknown
STIB 891	5.3±0.9	1.7±1.4	23.3±2.7	WT	WT	Unknown
DAL 870R	4.4±1.7	1.1±1.0	5.3±2.2	WT	WT	Relapse
DAL 898R	8.9±5.9	1.7±1.2	22.7±16.8	WT	WT	Relapse
K03048	24.8±9.2	81.2±21.9	58.0±33.6	WT	deletion/chimeric	Relapse
45 BT	25.9±8.6	91.8±29.7	37.5±10.8	WT	chimeric	Relapse
130 BT	42.3±17.6	76.9±22.3	12.3±4.5	WT	chimeric	Probable relapse
349 BT	26.2±11.3	71.9±12.4	20.0±3.2	WT	chimeric	Relapse
349 AT	25.6±11.8	81.9±31.8	15.4±1.0	WT	chimeric	Relapse
40 AT	22.0±8.0	72.2±21.1	39.9±16.7	WT	chimeric	Relapse
STIB 900	4.6±2.6	3.2±0.9	3.8±1.5	Ref	Ref	Cure
STIB 871	4.4±1.3	2.5±1.0	201±163	R allele	WT	Cure
STIB 940	13.6±7.0	3.4±2.0	340±218	R allele	WT	n.a.
STIB 950	27.6±9.4	1.8±0.4	102±53.6	R allele	WT	n.a.

WT = identical to reference (Ref) strain, being STIB 930 for *T. b. gambiense* isolates and STIB 900 for *T. b. brucei* and *T. b. rhodesiense* strains.

### Phenotyping

Drug sensitivity was determined with the Alamar blue assay as described by Räz et al (1997) [40], using the redox-sensitive dye resazurin as an indicator of cell number and viability. The trypanosomes were cultivated in 96-well microtiter plates in serial dilutions of drugs for 70 h. 10 ul of resazurin (125 ug/ml (Sigma) dissolved in PBS pH 7.2) was added to each well. The plates were further incubated for 2–4 hours for *T. b. rhodesiense* and *T. b. brucei*, and 6–8 hours for *T. b. gambiense*, before being read with a SpectraMax Gemini XS microplate fluorescence scanner (Molecular Devices) at an excitation wavelength of 536 nm and an emission wavelength of 588 nm. IC_50_ values were calculated by non-linear regression to a sigmoidal inhibition curve using SoftMax Pro software (V. 5.2). The IC_50_ values given in Table 2 are averages ± standard deviation of at least 3 independent assays (n = 3–12), each determined in duplicate. Melarsoprol (Sanofi-Aventis) was obtained from WHO. Pentamidine isothionate and diminazene aceturate were purchased from Sigma.

### Genotyping

Genomic DNA was isolated from 10 ml dense trypanosome cultures. The cells were spun down and the pellets resuspended in 300 µl 10 mM TrisHCl pH 8, 1 mM EDTA and 3 µl 10% SDS was added before incubating for 10–15 min at 55°C. After 5 min incubation 3 µl of pronase mix (20 mg/ml, Sigma) was added to increase the stability of the extracted DNA. 90 µl of ice cold 5 M potassium acetate was added and the mixture was incubated for 5 min on ice. After spinning down for 5 minutes at max speed in a microfuge, the supernatant was transferred to a new tube and DNA was precipitated in 2–2.5 volumes of absolute ethanol, washed in 70% ethanol and dissolved in 20 µl ddH_2_O. PCR was performed with Taq polymerase (Solis BioDyne, Estonia); the primers and annealing temperatures are summarized in Table S1. PCR products were run on a 0.8% agarose gel and purified on a silica membrane column (Nucleospin gel and PCR clean up, Macherey Nagel, Germany). The purified PCR products were directly sequenced (Microsynth, Switzerland or GATC, Germany) with the same primers as used for PCR amplification. Only the *TbAQP2/TbAQP3* locus of *T. b. gambiense* K03048 produced two PCR products, which were cloned in pCR2.1-TOPO (Invitrogen). The assembled sequences were submitted to GenBank; accession numbers are listed in Table S2.

## Results

### A panel of *Trypanosoma brucei* ssp. field isolates

To be able to compare – and possibly correlate – genotype and phenotype of *T. brucei* ssp., we assembled a set of 16 isolates that had been adapted to axenic *in vitro* cultivation as blood-stream forms. These included 5 recent *T. b. gambiense* isolates from the Democratic Republic of the Congo (DRC), 2 older isolates from the Republic of Côte d'Ivoire and one isolate from South Sudan, which were all isolated from patients who had relapsed after melarsoprol chemotherapy. Other *T. b. gambiense* isolates from the DRC, northwestern Uganda, and Liberia were from patients who were successfully treated with melarsoprol or the treatment outcome is unknown. *T. b. gambiense* STIB 930 is a fully drug-susceptible lab strain that was used as a reference strain. We further included the field isolates *T. b. brucei* STIB 940, *T. b. brucei* STIB 950 and *T. b. rhodesiense* STIB 871, which are multidrug-resistant to isometamidium, diminazene and tubercidin. The fully drug-susceptible reference strain *T. b. rhodesiense* STIB 900 was included as a reference. The different isolates and their origin are summarized in Table 1. All isolates were genotyped regarding *TbAQP2* and *TbAT1*.

### Naturally occurring mutations in *TbAQP2*

**Figure 1:**
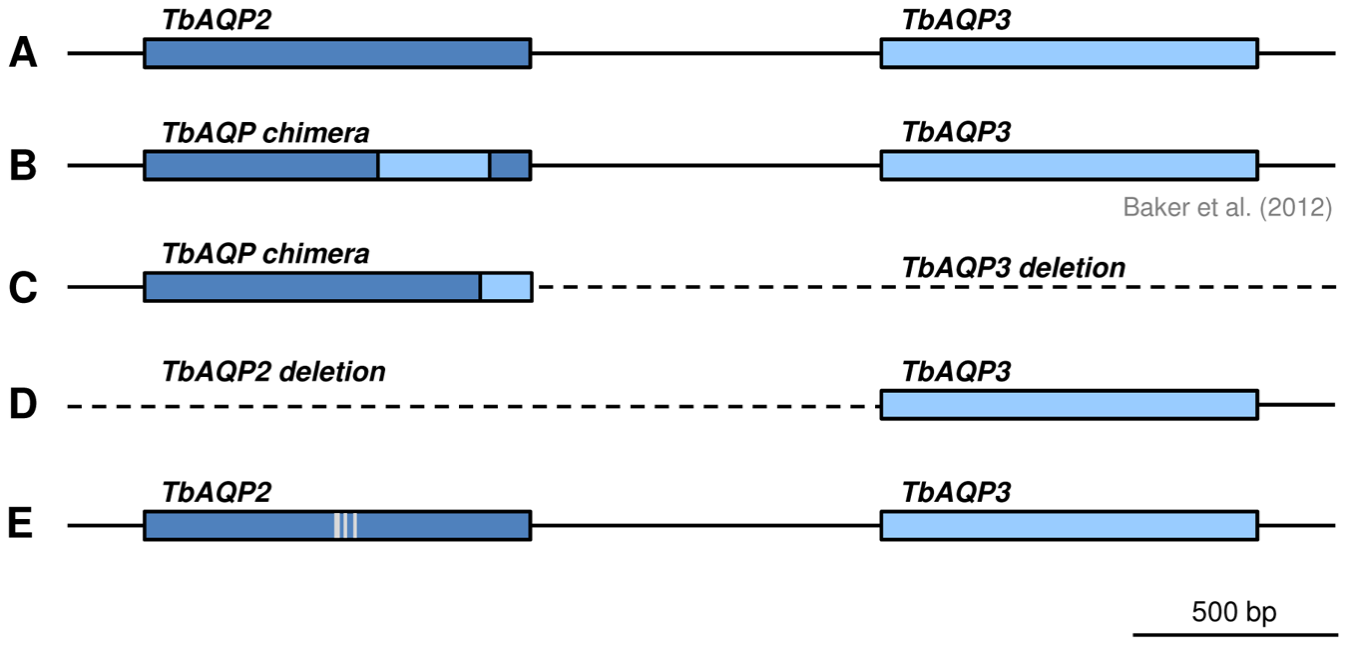
Schematic view of the *TbAQP2/TbAQP3* locus on chromosome 10. A) Reference locus of *T. b. brucei* TREU927, *T. b. gambiense* STIB 930 and *T. b. gambiense* DAL972 (minor differences in *TbAQP3* are not highlighted). B) Chimera of *TbAQP2* and *TbAQP3* as described by Baker et al. (2012) [24] for the *in vitro* selected, pentamidine-resistant *T. b. brucei* line B48. C) Chimera of *TbAQP2* and *TbAQP3* plus loss of *TbAQP3* in *T. b. gambiense* 40 AT, 45 BT, 130 BT, 349 BT, and 349 AT, and in one K03048 allele. D) Deletion of the *TbAQP2* ORF in the other *T. b. gambiense* K03048 allele. E) *TbAQP2* polymorphisms (C474A, G475A, C477T, T480C) in several *T. b. rhodesiense* and *T. b. brucei* isolates from East Africa (STIB 900, STIB 950, STIB 940, and STIB 871).

When the *TbAQP2/TbAQP3* genomic locus was amplified by PCR from the 16 *T. brucei* ssp. isolates, all the recent *T. b. gambiense* isolates from the DRC (40 AT, 45 BT, 130 BT, 349 BT and 349 AT) exhibited a smaller band than expected for the wild-type locus. Direct sequencing of the PCR product in each of the five isolates revealed only one gene at the locus: a chimeric version of *TbAQP2* and *TbAQP3*. The first 813 bp of the open reading frame perfectly matched *TbAQP2* while the remaining 126 bp derived from *TbAQP3* (Figure 1C). These 126 bp perfectly matched to *TbAQP3* of *T. b. rhodesiense* STIB 900 but this exact sequence is not found in the published genome of *T. b. gambiense* DAL 972. Note that the present *TbAQP2-TbAQP3* chimeric gene (Figure 1C) differs from the one described by Baker et al. from a pentamidine-selected *T. b. brucei* lab mutant (Figure 1B; [24]). *T. b. gambiense* K03048 from the South Sudan also gave rise to an abnormal pattern upon PCR amplification of the *TbAQP2/TbAQP3* locus from genomic DNA: a distinctly smaller double band instead of the expected product, indicative of heterozygosity. The smaller band contained the upstream region of *TbAQP2* followed by the open reading frame of *TbAQP3* while the *TbAQP2* open reading frame was missing (Figure 1D). The larger band contained a *TbAQP2/3* chimera similar to that encountered in the *T. b. gambiense* isolates of the DRC (Figure 1C). Point mutations in *TbAQP2* were encountered in the multidrug-resistant field isolates *T. b. brucei* STIB 940, *T. b. brucei* STIB 950 and *T. b. rhodesiense* STIB 871, all of which had the same 4 SNPs in *TbAQP2* compared to the *T. b. brucei* 927 reference gene (Tb927.10.14170), leading to the amino acid change threonine^159^ to alanine (Figure 1E). However, the same 4 SNPs also occurred in our drug-susceptible reference strain *T. b. rhodesiense* STIB 900, so they are not likely to be involved in the *mdr* phenotype [41], [42] of these isolates. All other isolates analyzed had a wild-type copy of *TbAQP2*. The identified sequence polymorphisms are summarized in Table 2, GenBank accession numbers are in Table S2.

### Naturally occurring mutations in *TbAT1*

All of the 12 analyzed *T. b. gambiense* isolates were identical in *TbAT1* sequence to the reference STIB 930 as well as to the genome strain DAL972. The previously described *TbAT1^R^* allele [19], [33] was found in the 3 *mdr* lines *T. b. brucei* STIB 940, *T. b. brucei* STIB 950 and *T. b. rhodesiense* STIB 871. *TbAT1^R^* carries 5 coding and 4 silent mutations and a codon deletion as compared to the reference sequence (STIB 900), and the resultant protein appeared to be non-functional when expressed in *Saccharomyces cerevisiae* [19] or re-expressed in a *tbat1* null *T. b. brucei* (De Koning, unpublished results). The remainder of the isolates did not possess mutations in *TbAT1* when compared to the respective reference isolate. The GenBank accession numbers of all the sequences are in Table S2.

### Correlating *TbAQP2* and *TbAT1* genotype to drug susceptibility

**Figure 2:**
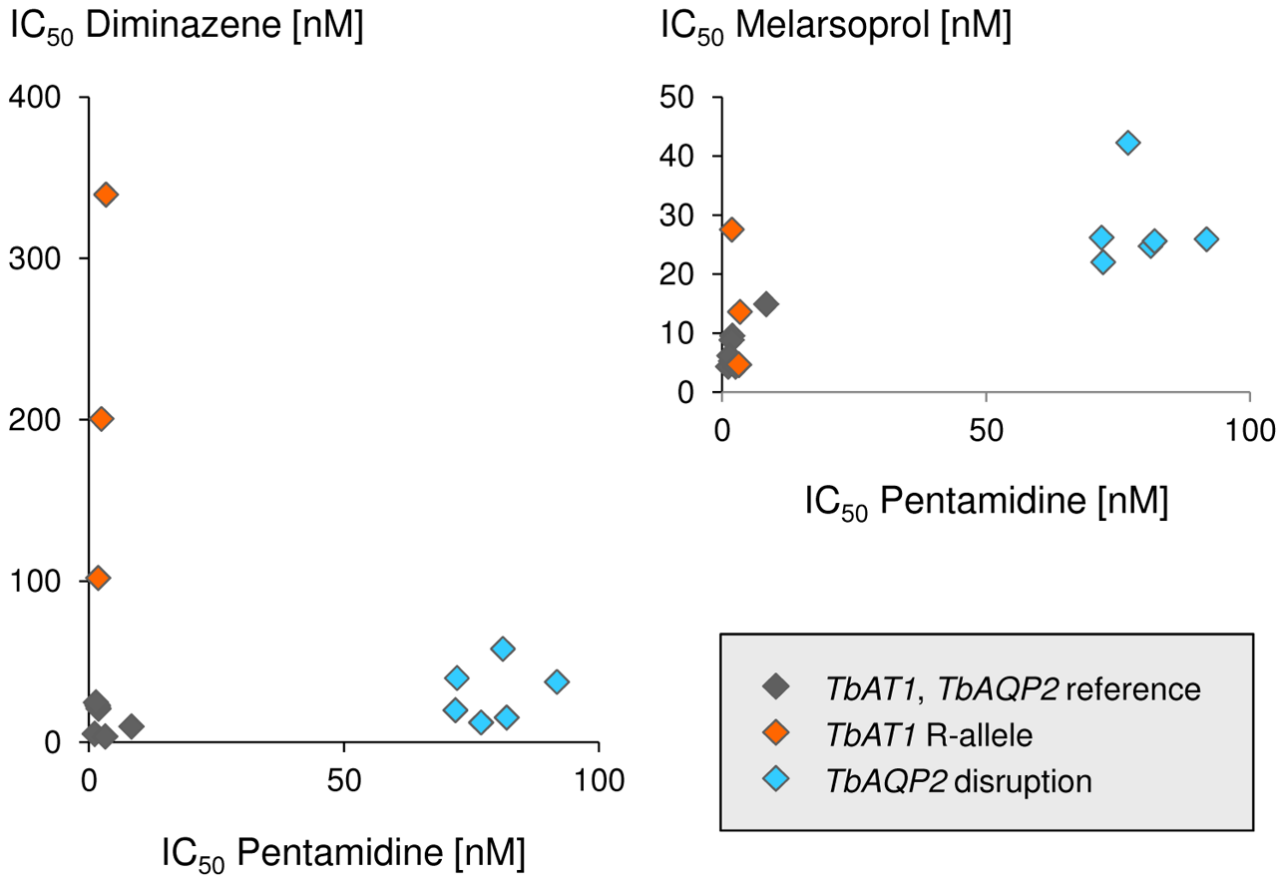
*In vitro* drug sensitivities. 50% inhibitory concentrations (IC_50_) as determined with the Alamar blue assay. Susceptibility to pentamidine correlates with that to melarsoprol but not diminazene. *TbAT1* and *TbAQP2* genotypes are indicated.

Drug sensitivities of the bloodstream-forms of all isolates were determined *in vitro* regarding melarsoprol, pentamidine, and diminazene. The five *T. b. gambiense* that possessed the chimeric *TbAQP2/3* gene (45 BT, 130 BT, 349 BT, 349 AT, 40 AT), as well as K03048 which carries a deletion of *TbAQP2* in one allele, in addition to one chimeric *TbAQP2/3* allele, all showed a similar drug sensitivity profile with markedly increased IC_50_ values towards pentamidine and, to a lesser extent, also melarsoprol (Figure 2). IC_50_ values were in the range of 70–92 nM for pentamidine and 22–42 nM for melarsoprol (Table 2); compared to the median of the four drug sensitive *T. b. gambiense* lines STIB 930, STIB 891, STIB 756 and ITMAP 141267, this corresponds to a 40- to 52-fold decrease in susceptibility to pentamidine and a 2.8- to 5.3-fold decrease for melarsoprol. The higher IC_50_ values of the isolates that carried a mutation in *TbAQP2* (n = 6) compared to the remainder (n = 10) were statistically significant both with respect to pentamidine (p = 0.0002, two-tailed Mann-Whitney test) and melarsoprol (p = 0.0047); no association was observed regarding *TbAQP2* status and sensitivity to diminazene. However, the isolates that carried the known resistance allele *TbAT1^R^* (i.e. STIB 940, STIB 950 and STIB 871) exhibited strongly increased IC_50_ values to diminazene (p = 0.01, two-tailed Mann-Whitney test) but not to pentamidine (Figure 2, Table 2). *T. b. brucei* STIB 950 also had an elevated IC_50_ against melarsoprol (Figure 2), but over all three *TbAT1^R^* isolates there was no significant effect on melarsoprol susceptibility.

Across all 16 *T. brucei* isolates, pentamidine sensitivity positively correlated with that to melarsoprol (Spearman's rank correlation coefficient of 0.67, p = 0.005) while there was no correlation between the two structurally related diamidines, pentamidine and diminazene (Figure 2).

## Discussion

It is an intriguing phenomenon with African trypanosomes that drug resistance is predominantly linked to reduced drug import, typically arising from loss of function mutation of a non-essential transporter [12], . Here we investigated the aminopurine transporter TbAT1 and the aquaglyceroporin TbAQP2, two proteins known to be involved in uptake of – and susceptibility to – melarsoprol and diamidines in bloodstream-form *T. brucei*. While there is evidence for a link between *TbAT1* mutations and melarsoprol treatment failure in the field [33]–[36], the more recently identified gene *TbAQP2* has so far not been analyzed in a clinical setting. *TbAQP2* is dispensable for growth in culture [24] and partial gene replacement of *TbAQP2* with *TbAQP3* was observed in a pentamidine-selected *T. b. brucei* lab mutant [24] that displayed reduced infectivity to rodents [21]. However, it was unknown whether similar mutations also occur in the field, as they might bear a fitness cost in patients or during transmission by the tsetse fly. Concentrating on a panel of clinical *T. brucei* ssp. isolates that (i) derived from treatment-refractory cases and (ii) had been adapted to axenic *in vitro* culture, we have genotyped their *TbAT1* and *TbAQP2* loci, and phenotyped their *in vitro* sensitivity towards melarsoprol, pentamidine and diminazene. Our aim was to explore whether *TbAQP2* mutations occur in the field and if so, whether mutant isolates exhibit reduced drug susceptibility.

Five of the analyzed *T. b. gambiense* isolates, all from melarsoprol relapse patients of Dipumba Hospital in Mbuji-Mayi, DRC, carried only one gene at the *TbAQP2*/*TbAQP3* tandem locus, an unprecedented *TbAQP2/3* chimera. The high degree of sequence similarity between *TbAPQ2* and *TbAQP3* allows for homologous recombination between the two genes, leading to chimerization and gene loss. TbAQP2 has a unique selectivity filter with unusual NSA/NPS motifs instead of the characteristic NPA/NPA that occur in the vast majority of MIP family members [43] including TbAQP1 and TbAQP3 [24]. The published, pentamidine-resistant *T. b. brucei* lab mutant possessed a TbAQP2/3 chimera whose C-terminal filter triplet was from TbAQP3, suggesting that the unusual NPS triplet may be involved in pentamidine transport. However, the presently described pentamidine-resistant *T. b. gambiense* isolates carry a *TbAQP2/3* chimera encoding a predicted protein with both selectivity filter triplets from TbAQP2. We hypothesize that the TbAQP2/3 chimera observed in the *T. b. gambiense* isolates fails to contribute to pentamidine and melarsoprol susceptibility despite having the proposed selectivity filter residues of TbAQP2. Functional expression of the chimeric gene in *tbaqp2* null cells will be necessary to test this hypothesis.

The occurrence of rearrangements at the *TbAQP2/TbAQP3* locus correlated with reduced susceptibility to pentamidine and, to a lesser extent, melarsoprol. Thus field isolates also exhibit the well known cross-resistance between melarsoprol and pentamidine 15,16,31, while no cross-resistance was observed to diminazene aceturate. This is in agreement with *TbAT1* being the primary uptake route for diminazene [44], [45] and consistent with results obtained using *TbAQP2* ^−/−^ cells, which showed no resistance to the rigid diamidines diminazene or DB75 [24], as opposed to pentamidine which has a highly flexible structure. It is also noteworthy that *T. b. rhodesiense* STIB 871 and *T. b. brucei* STIB 940 are susceptible to melarsoprol and pentamidine *in vitro* although both carry the *TbAT1^r^* allele. Loss of TbAT1 function has been described without mutations in the open reading frame of the gene [32]. However, since in the present study all isolates with a ‘wild-type’ *TbAT1* ORF were fully susceptible to diminazene, we conclude that they possess a functional TbAT1 (i.e. P2) transporter. *Trypanosoma congolense* and *T. vivax* appear to lack an AT1 orthologue [46], therefore diminazene transport and resistance must have a different mechanism in these livestock parasites.

The plasma levels of pentamidine in treated patients peak about 1 hour after injection and vary extensively from 0.42 µM to 13 µM, while the mean elimination half-life after multiple applications is approximately 12 days [47]. Thus, since pentamidine is very potent, even a 50-fold increase in IC_50_ of pentamidine as observed here for the *T. b. gambiense* isolates with mutations in *TbAQP2*, is unlikely to jeopardize the success of treatment. With melarsoprol, however, the obtainable drug levels are more critical. Only 1–2% of the maximal plasma levels are seen in the CSF [48], and a 5-fold reduced sensitivity to melarsoprol might allow trypanosomes to survive in the CSF during melarsoprol therapy. Thus mutations in *TbAQP2* might indeed be responsible for melarsoprol treatment failures with *T. b. gambiense*. However, two of the *T. b. gambiense* isolates from relapse patients (DAL 870R and DAL 898 R) were sensitive to melarsoprol and pentamidine, and they possessed wild-type copies of *TbAT1* and *TbAQP2*, indicating that factors other than drug resistance can contribute to treatment failures. Larger sample sizes will be required to test the significance of *TbAQP2* for successful treatment. We show here for the first time that a *TbAQP2/3* chimera as well as loss of *TbAQP2* occurs in *T. b. gambiense* clinical isolates, and that the presence of such rearrangements at the *TbAQP2/TbAQP3* locus is accompanied by a 40- to 50-fold loss in pentamidine sensitivity and a 3- to 5-fold loss in melarsoprol sensitivity. We recommend genotyping of the *TbAQP2*/*TbAQP3* locus to be integrated into larger field trials such as clinical studies with drug candidates.

## Supporting Information

Table S1 Primers used for PCR, their target gene, annealing temperature and sequence (5′ to 3′).
(PDF)

Table S2 GenBank accession numbers of the sequenced genes.
(PDF)
