# Supplementary material for: Aquaporin 2 Mutations in Trypanosoma brucei gambiense Field Isolates Correlate with Decreased Susceptibility to Pentamidine and Melarsoprol
Source: PLoS Negl Trop Dis. 2013 Oct 10;7(10):e2475. doi: 10.1371/journal.pntd.0002475 (PMC3794916; doi:10.1371/journal.pntd.0002475)
Supplement: Table S1 — Primers used for PCR, their target gene, annealing temperature and sequence (5′ to 3′). (PDF) [file pntd.0002475.s001.pdf]

Supplementary Table S1. Primers used for PCR.

**Target gene(s)**	**Gene ID TriTrypDB**	**Primer name**	**Annealing [°C]**	**Sequence**
Actin A	Tb09.211.0620<br>Tb09.211.0630	ACT_F	56	CCCTGAGTCACACAACGT
	Tb09.211.0620<br>Tb09.211.0630	ACT_R	56	CCCCAGCTGCATAACATT
TbAT1	Tb927.5.286b	TbAT1_F	56	GAAATCCCCGTCTTTTCTCAC
	Tb927.5.286b	TbAT1_R	56	ATGTGCTGAGCCTTTTTCCTT
	Tbg972.5.40	TbAT1_R_Tbg	56	ATGTGCTGACCCATTTTCCTT
TbAQP2	Tb927.10.14170	AQP2/3_F	58	AAGAAGGCTGAAACTCCACTTG
	Tb927.10.14170	AQP2_R	58	CTTCGGGAGAAACAAAACCTC
TbAQP3	Tb927.10.14160	AQP2/3_R	58	TGCACTCAAAAACAGGAAAAGA
