# Supplementary material for: Aquaporin 2 Mutations in Trypanosoma brucei gambiense Field Isolates Correlate with Decreased Susceptibility to Pentamidine and Melarsoprol
Source: PLoS Negl Trop Dis. 2013 Oct 10;7(10):e2475. doi: 10.1371/journal.pntd.0002475 (PMC3794916; doi:10.1371/journal.pntd.0002475)
Supplement: Table S2 — GenBank accession numbers of the sequenced genes. (PDF) [file pntd.0002475.s002.pdf]

Supplementary Table S2. GenBank accession numbers of the sequenced genes.

**Isolate**	***TbAT1***	***TbAQP2/TbAQP3***
STIB 930	KF564940	KF564925
ITMAP 141267	KF564946	KF564930
STIB 756	KF564945	KF564929
STIB 891	KF564944	KF564928
DAL 870R	KF564941	KF564926
DAL 898R	KF564942	KF564927
K03048	KF564943	
45 BT	KF564947	KF564931
130 BT	KF564948	KF564932
349 BT	KF564949	KF564933
349 AT	KF564950	KF564934
40 AT	KF564951	KF564935
STIB 900	KF564952	KF564936
STIB 871	KF564953	KF564937
STIB 940	KF564954	KF564938
STIB 950	KF564955	KF564939
